# Spatiotemporal Variations of the ^**90**^Sr in the Southern Part of the Baltic Sea over the Period of 2005–2010

**DOI:** 10.1155/2013/276098

**Published:** 2013-11-12

**Authors:** Michał Saniewski

**Affiliations:** Institute of Meteorology and Water Management, National Research Institute, Maritime Branch, Waszyngtona 42, 81-342 Gdynia, Poland

## Abstract

The Baltic Sea is one of the most contaminated seas by the radioactive isotope of strontium in the world; therefore the activity of ^90^Sr is regularly controlled. Due to that fact, seawater samples for ^90^Sr determination were collected at 16 stations located in the southern Baltic Sea between 2005 and 2010. In this period average activity of ^90^Sr was 7.8 Bq m^−3^ and varied within the range from 3.0 Bq m^−3^ to 11.9 Bq m^−3^. Because the higher activity of ^90^Sr was measured in the Baltic Sea than in the North Sea and rivers, inflows from the North Sea and the riverine runoff decreased ^90^Sr activity in the Baltic Sea. The average ^90^Sr activity in the bottom water along the offshore profile was 18% lower than that in the surface water and it was caused by an inflow of salt water from the North Sea. In the Vistula River mouth the average activity of ^90^Sr in the surface water was about 15% lower than the average activity in the bottom waters. Coastal areas, relatively shallow with good mixing condition in the water column, were characterized by low variability in ^90^Sr activity.

## 1. Introduction

Strontium occurs commonly in nature and has four natural stable isotopes, as well as sixteen unstable isotopes. ^90^Sr is of greatest importance as it is a byproduct of nuclear fission. Because of chemical similarity to calcium, ^90^Sr is built up in human bone, where it is retained for years and where its radiation might then cause deleterious effects on the health, such as leukemia or bone cancer [1]. Radioactive strontium is also dangerous for the organisms of benthic fauna and flora. ^90^Sr is one of the most hazardous radionuclides due to its long physical and biological half-life (28.8 years). For these reasons, the activity of ^90^Sr in the environment is monitored; however the complexity of the matrix components and incomplete recovery of the analytic cause the fact that radiochemical procedures used today are very complicated and expensive. The ^90^Sr activity is not often determined in environmental compartment [2–4]. The Baltic Sea is a brackish inland sea located in Northern Europe. Its saline water is a mixture of seawater from the North Sea and fresh water from the rivers and rainfall. The saltier water flowing from the North Sea does not easily mix with the less dense water in the Baltic Sea but sinks into the deeper basins. This causes in the Baltic Sea a strong density stratification. The open surface waters of the central basin have salinity of 6 to 8 PSU. In the Gulf of Finland and Gulf of Bothnia is lower (2 PSU). Below 40 to 70 m in the open Baltic Sea, the salinity is between 10 and 15 PSU and around 20 PSU near Danish Straits. The slow water exchange between the Baltic Sea and the North Sea ca. 30 years [5] leads to accumulation of toxic substances: hence it is also the case of radioactive isotope of strontium. The Baltic Sea is characterized by one of the highest concentrations of ^90^Sr in seawater, with only the Irish Sea and the Black Sea showing higher levels [6]. In 2000 the average activity of ^90^Sr in Iris Sea and Black Sea has been estimated to be 49 Bq m^−3^ and 17 Bq m^−3^, respectively. The main source of ^90^Sr in the Irish Sea was discharge from reprocessing plants at Sellafield [7]. The most important source of ^90^Sr in the Baltic Sea was global fallout from nuclear weapon tests carried out during the late 1950s and early 1960s, which consisted of 81% ^90^Sr in Baltic Sea [8, 9]. The Baltic Sea extends from 54′′ to 66′′ N which means that it lies within the zone of the most intensive global fallout. During the late 1990s the decay-corrected amounts of ^90^Sr from weapon test in the Baltic Sea have been evaluated at 500 TBq [6]. The second source of ^90^Sr, amounting to 13%, was the accident at the Chernobyl nuclear power plant in 1986. The total input of ^90^Sr from the Chernobyl accident over the Baltic Sea was estimated to be at about 80 TBq [10]. The third source of ^90^Sr to the Baltic Sea environment is the discharges from nuclear reprocessing sites in Western Europe (Sellafield and La Hague). The total discharge from these two nuclear power plants was estimated to be at 40 TBq (6% ^90^Sr) [10]. These latter sources are now only of minor importance, due to the significant reductions in discharges in recent years [6]. The additional sources of ^90^Sr were the nine nuclear power plants along the Baltic shores situated in Russia (Sosnovy Bor), Finland (Loviisa and Olkiluoto), Sweden (Forsmark, Barsebäck, Oskarshamn, and Ringhals), Lithuania (Ignalina, out of operation), and Germany (Greifswald, out of operation). Also a research reactor at Studsvik and Nuclear Fuel Factory of Westinghouse (ABB Atom), both located in Sweden, are potential sources of ^90^Sr [11]. In 1984–2010, the total input of ^90^Sr from all nuclear power plants and reactors in the Baltic Sea basin was about 1 TBq (0.2% ^90^Sr) [6, 11]. Nowadays, when nuclear weapon testing is limited and nuclear power plants are usually out of operation, the rivers and atmospheric deposition are the main sources of ^90^Sr to the Baltic Sea. The aim of the presented study was the examination of the level of ^90^Sr activity in sea water of the southern Baltic Sea and identification of factors affecting its activity. The southern Baltic Sea region is an area where both riverine water and inflows from the North Sea play significant roles in determining environmental conditions. That is why special attention was paid to the two processes.

## 2. Sampling and Analysis

**Figure 1:**
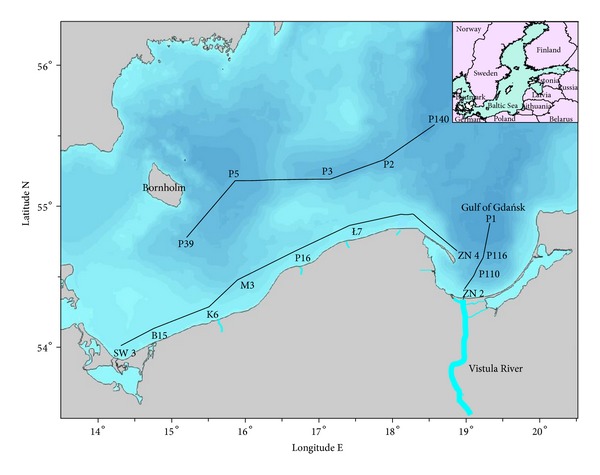
Localization of sampling stations.

Water samples were collected once a year in the period of 2005 to 2010 at the beginning of June. The sampling was done onboard R/V “Baltica” at 16 stations located in the southern Baltic Sea (Figure 1).

Seawater samples were taken with a rosette sampler, with simultaneous salinity and temperature profiling. At 11 stations (ZN2, P116, P110, L7, P16, M3, K6, SW3, B15, P2, and P3) surface water (0 m) and bottom water (2 m above the sea bottom) were collected. At five stations (P1, P140, ZN4, P39, and P5) water was collected along the water profile beginning from 0 m and at every 20 meters further down to 2 m above the sea bottom (Table 1).

**Figure 2:**
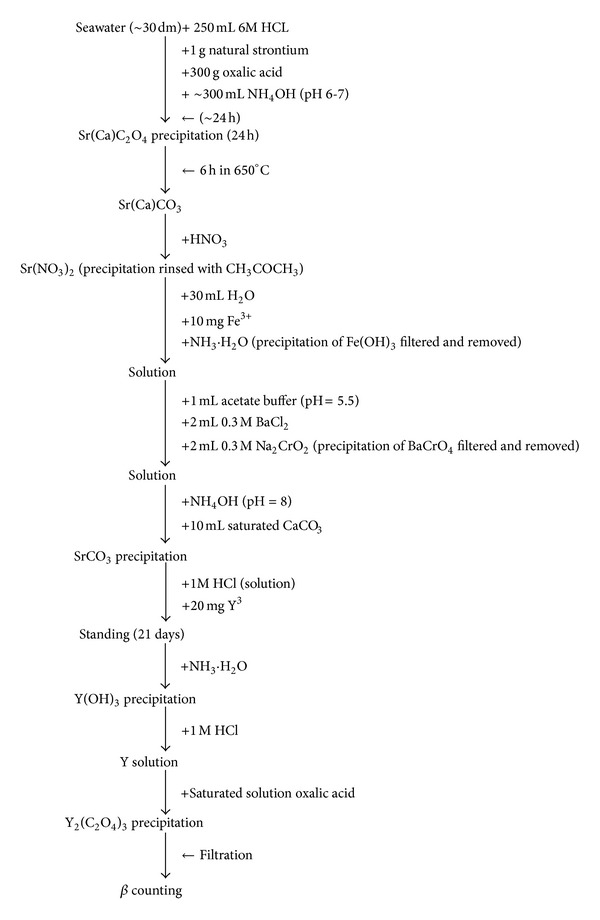
A flow chart of the analytical procedures used for ^90^Sr determination in seawater samples.

Seawater samples of ca. 30 dm^3^ volume were acidified (6 M HCl) immediately after sampling and transported to laboratory. One g of natural strontium was added to each sample (Figure 2). Strontium precipitated in the samples as oxalate during 10 min mixing (6 < pH < 7). After 24 h, the aqueous was decanted from the strontium oxalate precipitation. The oxalate was then converted to carbonates at 650°C. In the next analytical step strontium carbonate was separated from calcium carbonate with 65% HNO_3_. The removal of foreign ions, which can increase the activity of the final preparation, was carried out by the addition of Fe^3+^ in an alkaline medium, the Fe(OH)_3_ precipitating from the solution absorbs all ions which readily hydrolyze (Th^4+^, UO_2_^2+^, Ru^3+^, and Ce^3+^), and radium removal was done by precipitation with BaCrO_4_ in the presence of a buffering agent (pH = 5.5). 20 mg of stable yttrium was added, and the samples were allowed to stand for 21 days to allow secular equilibrium between ^90^Y and ^90^Sr to be attained [12]. Then yttrium was precipitated as hydroxide, converted to oxalate, and collected on a preweighed filter. Beta activity of samples was measured using Low-Level Beta Counter FHT 7700T (ESM Eberline) with the background count rate of 0.01 counts s^−1^ and the minimum detectable activity of 3 mBq per sample. In the period of 2005–2010, quality control of the laboratory analyses was ensured by participation in an international intercalibration exercise supervised by STUK, Radiation and Nuclear Safety Authority (Finland), concerning the analysis of ^90^Sr in seawater samples. The results fulfilled the criteria of quality and comparability of analytical results [13].

## 3. Results and Discussion

**Figure 3:**
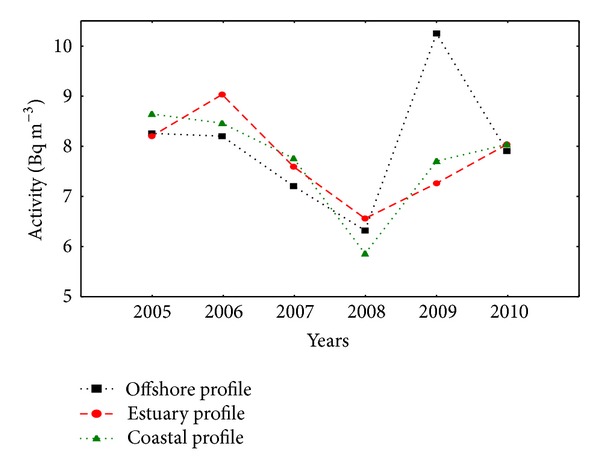
Average activities of ^90^Sr in sea water of the southern Baltic Sea.

In the years from 2005 to 2010, the average activity of ^90^Sr in sea water of the southern Baltic Sea was 7.8 ± 1.6 Bq m^−3^ and varied in the range from 3.0 Bq m^−3^ to 11.9 Bq m^−3^. These obtained results were comparable with the previous date and did not show a significant downward trend [14]. The lowest activity of ^90^Sr was recorded in the Gulf of Finland and Bothnian Bay, as reported by HELCOM [15], to be between 6 Bq m^−3^ to 8 Bq m^−3^. In Bornholm Basin, southern Baltic and northern Baltic Proper, Archipelago Sea, and the Bothnian Sea, ^90^Sr activity ranged from 9 Bq m^−3^ to 10 Bq m^−3^. Lower ^90^Sr activity was measured in rivers: the average activity of ^90^Sr in Vistula River in period of 2005–2010 was 5.4  ±  2.1 Bq m^−3^. In the North Sea activity of ^90^Sr ranged from 1.2 Bq m^−3^ to 3.7 Bq m^−3^ [16]. In North and South Atlantic average activity of ^90^Sr in surface water was 1.2 Bq m^−3^ and 0.4 Bq m^−3^, respectively [7]. Considering the impact of inflow water from North Sea and from rivers, the sampling stations were divided into three profiles: coastal, offshore, and estuarine. Average activities of ^90^Sr in the water in each profile varied according to hydrological and meteorological conditions and were discussed in the next sections (Figure 3).

### 3.1. The Offshore Profile

**Figure 4:**
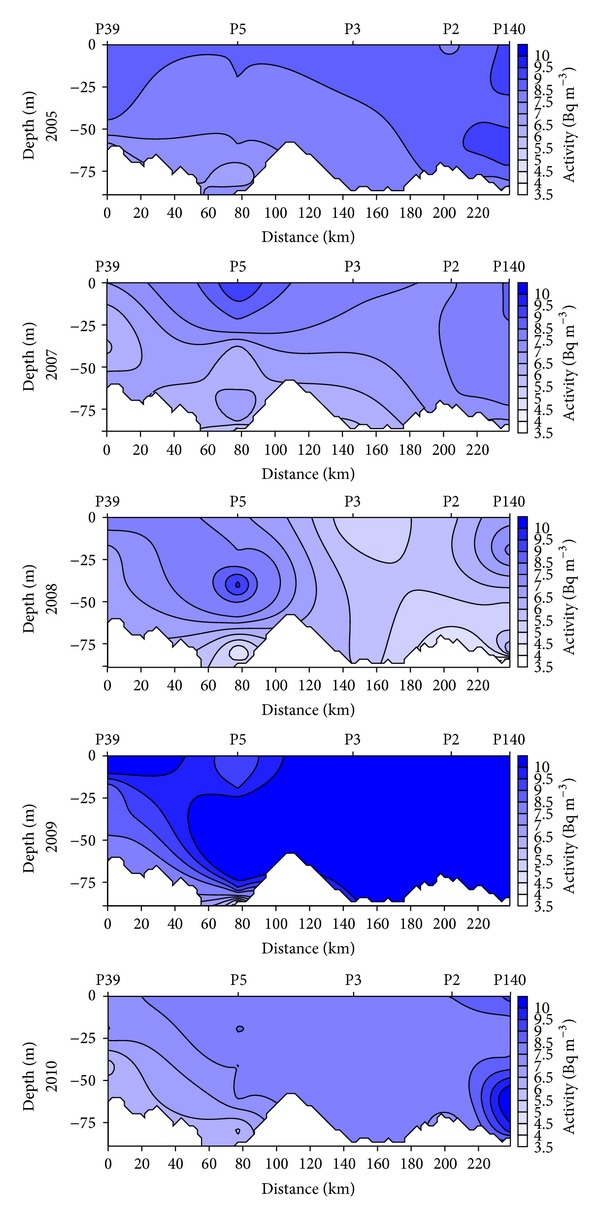
Seasonal and spatial variations in ^90^Sr activity in the offshore profile in the years of 2005–2010.

The average activity of ^90^Sr in surface water in the period of 2005–2010 was 8.5 ± 1.6 Bq m^−3^ and it was higher by 18% than the activity in the bottom water (7.0 ± 1.8 Bq m^−3^). Usually the concentration of ^90^Sr did not vary from the surface down to the upper limit of the halocline. Beneath the halocline, ^90^Sr activity declined rapidly with the increasing salinity. The lowest activity of ^90^Sr was recorded in the near bottom water (Figure 4). This observation is directly related to the inflow of salty water from the North Sea. Saltier water is introduced into the Baltic Sea to the bottom layers due to specific meteorological conditions. If the magnitude of the inflow is sufficient, the near bottom water moves along the Baltic deep basins and displaces the water residing there. This mechanism was especially important for the westernmost area of two stations P39 and P5 where the impact of saline water inflow was the biggest and the correlation between salinity and activity was statistically significant (*r* = −0.6513, *P* < 0.0001, *n* = 51). The lowest activity of ^90^Sr was measured in 2008 (6.3 ± 1.5 Bq m^−3^), when the long-lasting westerly winds contributed to the inflow of water from the North Sea. In June 2008, the highest salinity (15.84 PSU) was recorded in the Bornholm Deep (P5). The inflow of water from North Sea caused a significant decrease of the activity of bottom water to 5.1 ± 1.3 Bq m^−3^, lower by 28% than the average 7.0 ± 1.8 Bq m^−3^ from whole period of research. The highest activity of strontium 10.2 ± 1.7 Bq m^−3^ was recorded in 2009. The average activity in the entire water was higher by 22% than the average 8.0 ± 1.8 Bq m^−3^ from 2005 to 2010. The main reason for this increase was the exchange of waters from the northern Baltic Proper where the ^90^Sr activity was about 10 Bq m^−3^ [17]. In April 2009, a strong surface current from east was observed in surface waters of the central basin [18]. In the easternmost areas increase of activity in the entire water column to 11.4  ±  0.4 Bq m^−3^ was observed. At the same time lower salinity of bottom water was reported thus confirming the increase in activity. However, in the far western area (stations P39 and P5), where the strong salinity stratification occurred (bottom water was characterized by high salinity of about 13–16 PSU), the water flowing from the east with lower salinity was not able to displace bottom water. This resulted only in increase of activity at the upper layer of halocline.

### 3.2. Estuary Profile

**Figure 5:**
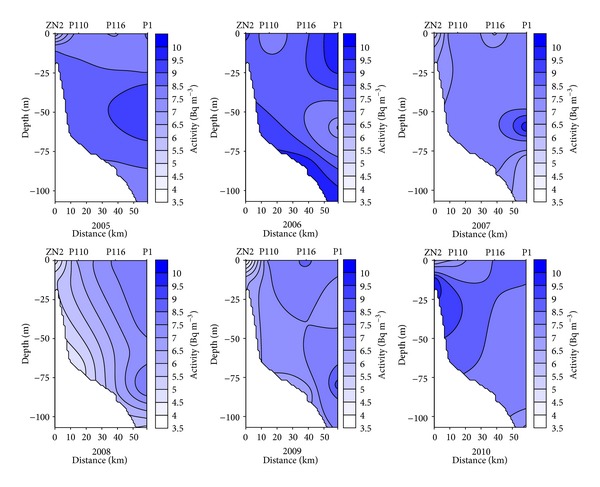
Seasonal and spatial variations in ^90^Sr activity in the estuarine profile in the years of 2005–2010.

The drainage basin of the Baltic Sea covers an area of about 4.3 times larger than the sea itself, bringing large amounts of freshwater to the sea from the rivers [19]. In the Baltic Sea catchment, the least significant source of ^90^Sr to the land was the atmospheric deposition after the Chernobyl accident. The total deposition from Chernobyl on land amounted to 10 PBq ^90^Sr and was 125 times more than got directly to the Baltic Sea, because the less volatile isotope was confined to areas closer to the damaged reactor and later washed out with rain and riverine outflow [7]. In the terrestrial environment strontium cations are mobile and easily washed out by rain water to the rivers and lakes [10, 20]. Nowadays, the water of the Vistula contains about 30% less ^90^Sr than water of the southern Baltic Sea. In the estuary profile (Figure 5) ^90^Sr activity is determined by two factors: the outflow of the Vistula and the inflow of water from the northern parts of the Baltic Sea. In this profile, which has effect opposite to offshore profile, the activity of ^90^Sr in water increased with salinity due to the diluting of the riverine water. The lowest strontium activity was recorded at the station in close proximity to the estuary and the highest one at the station located farthermost from the estuary. This is particularly marked at two measurement stations, located at a distance of ca. 2 km (ZN2) and 20 km (P110) from the river of Vistula mouth. A strong statistically significant correlation of ^90^Sr activity with salinity for these stations was found: *r* = 0.580 (*P* = 0.0047, *n* = 24). An exceptional situation was observed in 2010 when, due to heavy precipitation in May, a flood occurred in the Vistula River catchment area, and, as a result, a number of flood crests discharged into the Gulf of Gdańsk [21–23]. The fresh water outflow into the Baltic Sea during May and June 2010 amounted to 235% and 319%, respectively, of the long-term (1951–2000) mean. The correlation of ^90^Sr and salinity was then characterized by *r* = 0.639 (*P* = 0.0642, *n* = 10). However, the diluting effect of fresh water in the surface water layer of the Gulf of Gdańsk was not so obvious as in previous years because of the relatively high activity of strontium in riverine water, reaching 5.8 Bq m^−3^ (Figure 5). The average activity of ^90^Sr in surface water at a station of the 20 km distance from the river mouth reached then 6.0 ± 1.6 Bq m^−3^ and it was lower by 15% from the activity determined in near bottom water (7.6 ± 1.6 Bq m^−3^). An example of differing picture of ^90^Sr activity distribution—with negligible difference between the surface and near bottom water—was found out in 2008, when, as a result of heavy storm, intensive marine currents caused strong mixing in the entire water column and vary similar values (ca. 5 Bq m^−3^) of strontium activity in surface and near bottom water were determined at both analyzed stations in the Gulf of Gdańsk. The second factor determining the radioactivity in the Gulf of Gdańsk was the influx of water from the northern part of the Baltic Sea, where surface activity of ^90^Sr in the surface water is higher by approximately 25% (9.9 Bq m^−3^) than that in the Gulf of Gdańsk (7.5 Bq m^−3^) [17, 24]. In 2006, the activity of ^90^Sr in the surfer water (9.0 Bq m^−3^) was higher by 18% than average activity in this profile in the period of 2005–2010 (7.5 Bq m^−3^) due to smaller riverine outflow and the dominating winds from the NE sector (25%) [25]. Such distribution of winds pushed the river water into the eastern part of the gulf. At the closest measurement station in the closest proximity to the river mouth (ZN2), the activity compared to that of the preceding year was almost twice as high (9.2 Bq m^−3^). This was also confirmed by salinity which in 2006 was 6.0 PSU, and it was about 3 PSU higher than that in 2005 (Figure 5).

### 3.3. Coastal Profile

**Figure 6:**
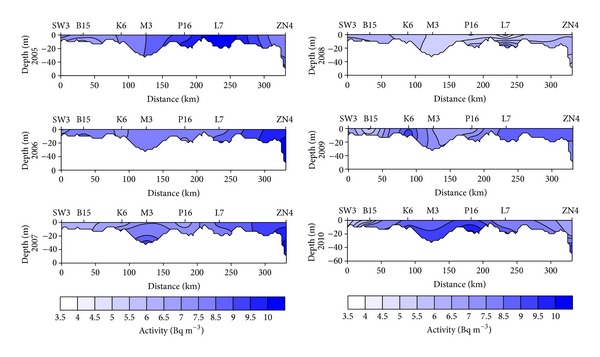
Seasonal and spatial variations in ^90^Sr activity in the coastal profile in the years 2005–2010.

The sampling stations at the coastal profile are shallow; that is, the mixing encompasses the entire water column and at the same time this profile is the least exposed to riverine water impact as well as to the salt water inflows from the North Sea. The hydrodynamic conditions in the coastal profile determine that the distribution of radioactive strontium in seawater is more uniform regarding both the horizontal and vertical profiles. The distance to input sources was also the reason for relatively low variability (SD = 1.5 Bq m^−3^) of ^90^Sr activity in seawater of this region. The activity of ^90^Sr in seawater of this region depended strongly on wind direction. Under wind from western direction, water was transported from western parts of the Baltic Sea, and as it was poorly supplied in ^90^Sr, the resulting concentrations were lower. Wind from eastern directions pushes water from the Baltic Proper close to the shore; hence the activity increased (Figure 6). At stations B13 and SW3, the farthest to the west, strontium activity in surface seawater was also influenced by the riverine discharge of fresh water from the Oder though the effect was not as conspicuous as in the case of Vistula. Despite the fact that ^90^Sr activity in water close to the Oder mouth was lower (6.8 ± 1.0 Bq m^−3^) by only ca. 8% than the activity in the entire profile (7.4 ± 1.4 Bq m^−3^), the statistically significant correlation between strontium activity and salinity was found here as well *r* = 0.231, *P* = 0.0162, and *n* = 113.

## 4. Conclusions

- The major factors influencing ^90^Sr activity in seawater of the Baltic Sea between 2005 and 2010 were the hydrological forces (riverine discharges of fresh water and salt water inflows from the North Sea) as well as meteorological conditions, mainly wind direction distribution.
- The average ^90^Sr activity in near bottom water in the offshore profile was lower by 18% than that in surface water layer, mainly due to diluting effect of salty water inflows from the North Sea.
- The impact of fresh water from the Vistula River plume shows only up to 20 km distance from the river mouth, and strontium activity in surface water here is lower by 15% than that in the near bottom water layer.
- Winds from the western directions cause decrease in ^90^Sr activity in seawater along the central Polish coast, because they push Sr-free water from the North Sea and western Baltic areas.
- Winds from the eastern directions result in an increase in the radionuclide concentrations as they transport Sr-rich water eastern Baltic Proper.

## Figures and Tables

**Table 1:** Coordinates and total depth of the sampling stations.

Station	Depth (m)	Latitude	Longitude	Profile	Baltic Sea region
P39	61	54°45′N	15°08′E	Offshore	Bornholm Basin
P5	87	55°15′N	15°59′E		Bornholm Basin
P3	89	55°15′N	17°04′E		Bornholm Basin
P2	75	55°17′N	18°00′E		Eastern Baltic Proper
P140	85	55°33′N	18°24′E		Eastern Baltic Proper

ZN2	12	54°23′N	18°57′E	Estuary	Gulf of Gdańsk
P110	69	54°30′N	19°01′E		Gulf of Gdańsk
P116	85	54°39′N	19°18′E		Gulf of Gdańsk
P1	105	54°50′N	19°20′E		Gulf of Gdańsk

SW3	8	53°57′N	14°16′E	Coastal	Bornholm Basin
B15	12	54°04′N	14°42′E		Bornholm Basin
K6	12	54°15′N	15°32′E		Bornholm Basin
M3	34	54°27′N	15°59′E		Bornholm Basin
P16	16	54°38′N	16°48′E		Bornholm Basin
L7	19	54°50′N	17°31′E		Eastern Baltic Proper
ZN4	69	54°40′N	18°50′E		Gulf of Gdańsk
